# The Purified Siderophore from *Streptomyces tricolor* HM10 Accelerates Recovery from Iron-Deficiency-Induced Anemia in Rats

**DOI:** 10.3390/molecules27134010

**Published:** 2022-06-22

**Authors:** Hassan Barakat, Kamal A. Qureshi, Abdullah S. Alsohim, Medhat Rehan

**Affiliations:** 1 Department of Food Science and Human Nutrition, College of Agriculture and Veterinary Medicine, Qassim University, Buraydah 51452, Saudi Arabia; 2 Department of Food Technology, Faculty of Agriculture, Benha University, Moshtohor 13736, Egypt; 3 Department of Pharmaceutics, Unaizah College of Pharmacy, Qassim University, Unaizah 51911, Saudi Arabia; ka.qurishe@qu.edu.sa; 4 Faculty of Biosciences and Biotechnology, Invertis University, Bareilly 243123, Uttar Pradesh, India; 5 Department of Plant Production and Protection, College of Agriculture and Veterinary Medicine, Qassim University, Buraydah 51452, Saudi Arabia; a.alsohim@qu.edu.sa (A.S.A.); m.rehan@qu.edu.sa (M.R.); 6 Department of Genetics, Faculty of Agriculture, Kafrelsheikh University, Kafr El-Sheikh 33516, Egypt

**Keywords:** *Streptomyces tricolor*, siderophore, purification, iron-deficiency-induced anemia

## Abstract

Iron-deficiency-induced anemia is associated with poor neurological development, including decreased learning ability, altered motor functions, and numerous pathologies. Siderophores are iron chelators with low molecular weight secreted by microorganisms. The proposed catechol-type pathway was identified based on whole-genome sequences and bioinformatics tools. The intended pathway consists of five genes involved in the biosynthesis process. Therefore, the isolated catechol-type siderophore (Sid) from *Streptomyces tricolor* HM10 was evaluated through an anemia-induced rat model to study its potential to accelerate recovery from anemia. Rats were subjected to an iron-deficient diet (IDD) for 42 days. Anemic rats (ARs) were then divided into six groups, and normal rats (NRs) fed a standard diet (SD) were used as a positive control group. For the recovery experiment, ARs were treated as a group I; fed an IDD (AR), group II; fed an SD (AR + SD), group III, and IV, fed an SD with an intraperitoneal injection of 1 μg Sid Kg^−1^ (AR + SD + Sid1) and 5 μg Sid Kg^−1^ (AR + SD + Sid5) twice per week. Group V and VI were fed an iron-enriched diet (IED) with an intraperitoneal injection of 1 μg Sid Kg^−1^ (AR + IED + Sid1) and 5 μg Sid Kg^−1^ (AR + IED + Sid5) twice per week, respectively. Weight gain, food intake, food efficiency ratio, organ weight, liver iron concentration (LIC) and plasma (PIC), and hematological parameters were investigated. The results showed that ~50–60 mg Sid L^−1^ medium could be producible, providing ~25–30 mg L^−1^ purified Sid under optimal conditions. Remarkably, the AR group fed an SD with 5 μg Sid Kg^−1^ showed the highest weight gain. The highest feed efficiency was observed in the AR + SD + Sid5 group, which did not significantly differ from the SD group. Liver, kidneys, and spleen weight indicated that diet and Sid concentration were related to weight recovery in a dose-dependent manner. Liver iron concentration (LIC) in the AR + IED + Sid1 and AR + IED + Sid5 groups was considerably higher than in the AR + SD + Sid1 AR + SD + Sid5 groups or the AR + SD group compared to the AR group. All hematological parameters in the treated groups were significantly closely attenuated to SD groups after 28 days, confirming the efficiency of the anemia recovery treatments. Significant increases were obtained in the AR + SD + Sid5 and AR + IED + Sid5 groups on day 14 and day 28 compared to the values for the AR + SD + Sid1 and AR + IED + Sid1 groups. The transferrin saturation % (TSAT) and ferritin concentration (FC) were significantly increased with time progression in the treated groups associatively with PIC. In comparison, the highest significant increases were noticed in ARs fed IEDs with 5 μg Kg^−1^ Sid on days 14 and 28. In conclusion, this study indicated that Sid derived from *S. tricolor* HM10 could be a practical and feasible iron-nutritive fortifier when treating iron-deficiency-induced anemia (IDA). Further investigation focusing on its mechanism and kinetics is needed.

## 1. Introduction

Iron, a trace metal, is crucial in maintaining normal human metabolism and is required by most organisms. It is used in numerous living processes, including oxygen transport, redox reactions, and nucleic acid synthesis [1,2]. Iron-deficiency-induced anemia (IDA) can result in various diseases, including delays in development and behavior in children and, in particular, in pregnant women [3,4,5,6]. Over two billion people worldwide suffer from IDA, according to the FAO (Food and Agriculture Organization) [3,7]. Iron-deficiency-induced anemia and latent iron deficiency significantly increase and spread among younger individuals [8]. Iron deficiency and IDA arise when dietary iron requirements cannot meet the physiological requirements of the human body, particularly in developing countries, primarily due to insufficient iron content in food and low iron absorption efficiency. Iron-deficiency-induced anemia causes tissue hypoxia, fatigue, headaches, and palpitations. Its association with neurological and psychiatric symptoms has been reported, as epidemiological evidence has shown that restless leg syndrome is associated with iron-deficiency-induced anemia [9]. Iron deficiency symptoms are not limited to anemia alone; for example, prolonged iron deficiency is known to cause inferior papillary atrophy, angular cheilitis, etc. [10]. Iron is found in animal and plant foods in two forms, i.e., heme and non-heme iron; however, non-heme iron constitutes approximately 90% of the iron absorbed from food [11]. Though iron is absorbed in the intestinal tract [12], non-heme iron is much more poorly absorbed than heme iron [13]. Therefore, it is necessary to make iron absorption more efficient and supplement daily diets with iron.

Siderophores are low-molecular-weight compounds (200–2000 Da) secreted by microbes, fungi, and plants under limited iron conditions [14]. Under iron-deficient growth conditions, the hydrophobicity of microbial surface decreases; then, the surface protein composition will alter, leading to the limitation of biofilm formation [15]. When iron becomes unavailable in the environment, microorganisms start producing siderophores as a developed specific uptake strategy. Siderophores consider a metal-chelating agent that can be produced especially under Fe-limiting conditions, making them available for microorganisms [16,17]. They could facilitate the iron acquisition and counter iron deficiency by high-affinity iron (III) ligands [14]. Compared with other metal elements, iron is preferentially chelated by siderophores [18].

Moreover, ferric iron considers the only state that siderophores could chelate. When ferric iron is reduced to ferrous iron, siderophores will release it. This is an important strategy for microbes to combat the iron stress environment [14,19]. The role of siderophores in microbial physiological activities is to dissolve ferric iron and transport it into the cytoplasm, so siderophores have the potential to become a nutritional fortifier [20]. The majority of marine siderophores have distinctive structural features [14]. Accordingly, they contain amphipathic and photochemical properties in Fe^3+^ complexes. Depending on membrane affinity and length, siderophores could anchor a particular gradient outside the cell membrane, thereby enhancing the ability to capture iron from seawater [18,21].

A siderophore is a biological molecule with many applications, and various bacteria can produce it. It can be utilized in multiple fields such as agricultural, medicinal, and environmental applications [22,23,24]. In therapeutic applications and iron-overload diseases (hemosiderosis, β-thalassemia, hemochromatosis, and accidental iron poisoning), iron is needed in the body so it can be treated with siderophore-based drugs [23,24]. Because of their rapid cell division, cancer cells have a higher requirement of iron when compared to healthy cells. Additionally, their iron uptake and storage rates are higher, so iron chelators such as siderophores can be beneficial for use in cancer therapy (iron excess or iron overload). It was shown that desferrioxamines (hydroximate-type siderophores) significantly decreased tumor cell growth in patients with neuroblastoma or leukemia [25,26]. Furthermore, desferrioxamine E produced by *Streptomyces* reduced the viability of malignant melanoma cells significantly. At the same time, other siderophores (i.e., desferriexochelins, desferrithiocin, tachpyridine, O-trensox, and dexrazoxane) were used in cancer therapy as iron chelators [27,28].

The iron-conveying abilities of siderophores are used to deliver drugs. Drug delivery was shown to occur in cells by forming conjugating among antimicrobials such as albomycins and siderophores [29]. Siderophores can be potentially used as iron chelators in treating cancers, e.g., dexrazoxane, desferriexochelins, desferrithiocin, and *O*-trensox. Furthermore, siderophores can rescue non-transferrin-bound iron in blood serum [29]. Siderophores produced by *Klebsiella pneumoniae* can act as antimalarial agents and can be used to treat malaria caused by *Plasmodium falciparum* [30].

*Streptomyces*, Gram-positive bacteria, are famous for producing various secondary metabolites. These secondary metabolites have significant biological and clinical usages. Secondary metabolite biosynthesis is usually controlled by complex regulatory networks that respond to various biotic and abiotic stresses in the surrounding environment. *Streptomyces* are considered to be one of the most common producers of secondary metabolites (i.e., antibiotic, antibacterial, antifungal, anticancer, siderophores, etc.) [31,32,33,34].

Siderophores could potentially become exciting molecules for combating IDA. However, despite the literature showing promising potentialities related to siderophores, particular attention needs to be paid to the applicability, dose, and mechanisms of siderophores. Moreover, to the best of the authors’ knowledge, the literature review mainly highlighted the purification and applications of siderophores, with only a few studies referring to in vivo applications [35,36]. The need to further verify siderophore applications motivated this work. Interestingly, the iron chelation capability of siderophores concerning malignant cancerous cells has been recently studied [37]. Suehiro et al. [38] proved that hemoglobin and hematocrit levels were significantly increased with maltobionic acid calcium salt (MBCa) intake, and recovery from iron-deficiency-induced anemia was promoted. MBCa effectively promoted the recovery of rats from subclinical iron deficiency and iron-deficiency-induced anemia. Feng et al. [35] indicated that siderophores derived from *Synechococcus* sp. PCC7002 could be practical and feasible iron-nutritive fortifiers. However, no studies have clearly demonstrated the effectiveness of using siderophores to enhance iron absorption in human food models. In anemia conditions, a lack of sufficient healthy red blood cells to carry adequate oxygen to the body’s cells and low hemoglobin content causes fatigue and weakness. Iron-deficiency-induced anemia is the most common type of anemia worldwide, caused by a shortage of iron in the body. Bone marrow requires iron to produce hemoglobin. When iron levels are low, our bodies cannot produce enough hemoglobin for red blood cells. Therefore, the main objective of this study was to study the potential application of purified Sid from *S. tricolor* HM10 bacteria to accelerate recovery from iron-deficiency-induced anemia in a rat model.

## 2. Materials and Methods

### 2.1. Siderophore, Detection, Production, and Purification from S. tricolor HM10

*S. tricolor* HM10 strain (accession: MN527236) was used for siderophore production, as previously established by Rehan et al. [39], with minor modifications. For siderophore detection, the Chrome Azurol S (CAS) assay was applied by adding CAS solution prepared according to Schwyn and Neilands [40] to King’s B agar medium (composed of peptone, 20.0; K_2_HPO_4_, 1.50; MgSO_4_.7H_2_O, 1.50; agar, 15.0 (g/L), pH 7.2) with a ratio of 1:9. In Sid production, a fresh culture of *S. tricolor* HM10 was cultivated on King’s B medium supplemented with FeCl_3_ (5 μM) for 72 h at 28 °C with continuous agitation (170 rpm min^−1^). Afterward, the culture broth was centrifugated at 10,000× *g* for 20 min^−1^. Sid concentration and type were determined in clear supernatant using Arnow’s Assay [41] for catechol-type siderophore and Atkin’s assay [42] for hydroxamate-type siderophore. According to Clark [43], after the acidification of the collected supernatant to pH 2.0 using concentrated HCl, the Sid produced was isolated and purified. The culture supernatant was passed through a column (5 × 30 cm) packed with Amberlite XAD-2, Sid was eluted with methanol, and fractions were analyzed for the presence of Sid with Arnow’s Assay. According to their hydrophobicity, the collected fractions were loaded on the Sephadex LH-20 column and eluted with methanol as the mobile phase for separation. Subsequently, TLC was used to test the collected fractions with Sid activity using a mobile phase containing methanol: ammonium acetate (60:40) [44]. Before injecting the solutions into rats, the concentration of purified Sid was determined with a standard curve generated by 2,3-Dihydroxybenzoic acid (2,3-DHBA). The injected solutions were freshly prepared at two concentrations (1 μg and 5 μg Sid) in sterilized saline buffer (pH 7.0).

### 2.2. Whole-Genome Sequence Analysis

The whole genome of *S. tricolor* HM10 was sequenced using Oxford Nanopore, assembled, and annotated. The genome is available in the NCBI database with accession number JAJREA000000000. In terms of searching the entire genome, the catechol-type pathway putatively coding for siderophores close to petrobactin was identified using blast search and antiSMASH [45,46].

### 2.3. Determination of Total Phenolic Content (TPC) and Antioxidant Activity (AOA) of Purified Sid

The TPC-purified Sid was determined by using Folin–Ciocalteu reagent according to Yawadio Nsimba et al. [47], and the TPC content was expressed as milligrams of Gallic acid equivalents (GAE) per gram (mg of GAE g^−1^ DW). The radical scavenging activity was measured spectrophotometrically based on the bleaching of DPPH radicals in purple solution according to Yawadio Nsimba et al. [39], and ABTS (2,2′-azino-bis (3-ethylbenzothiazoline-6-sulphonic acid)) radicals in a dark green solution using the method by Barakat and Rohn [48]. The final results were expressed as millimoles of Trolox Equivalents (TE) per gram (mmol of TE g^−1^).

### 2.4. Bio-Evaluation of Iron Absorption Using a Rat Model

#### 2.4.1. Experimental Animals

The study protocol was approved by the Institutional Animal Ethics Committee (IAEC) of Qassim University, KSA (21-07-06 on 26 January 2022). It was regulated by the Purpose of the Control and the Supervision of Experiments on Animals (CPCSEA) Committee under the National Committee of Bioethics (NCBE), Implementing Regulations of the Law of Ethics of Research on Living Creatures. Fifty-six female Wistar rats (6–8 weeks) weighing 150–200 g were housed in polypropylene laboratory cages (four each) in a room at 24 ± 1 °C under inverted 12 h light–dark cycle conditions. Before the feeding experiment, the rats were allowed to adapt to the AIN-93G standard diet (SD) for one week, as Suehiro et al. [38] recommended, as shown in Table 1.

#### 2.4.2. Experimental Diets

Table 1 shows the diet compositions utilized during the anemia induction and recovery periods. An iron-deficient diet (IDD) was created during the anemia-inducing period by slightly altering the AIN-93G diet used during the adaptation period. The rats were divided into two groups: the first (8 rats) were fed an SD for 42 days, and the second (48) were provided an IDD for 42 days. According to Suehiro et al. [38], rats administered the IDD had their serum iron level measured randomly and were deemed anemic if their plasma iron concentration (PIC) was less than 50 g dL^−1^. The animals were given unlimited access to deionized water. Anemic rats were separated into six groups during the anemia recovery period, each having eight rats. Anemic rats (ARs) in group I was fed an IDD for another four weeks. Group II (AR + SD) received the SD, group III (AR + SD + Sid1) received the SD and received purified Sid at 1 μg Kg^−1^ rat twice per week, and group IV (AR + SD + Sid5) received the SD and received pure Sid at 5 μg Kg^−1^ rat twice each week. The iron-enriched diet (IED) was created with double the iron content of the standard diet (SD) and administered to groups V and VI. Group V (AR + IED + Sid1) received pure Sid intraperitoneally twice a week at a dose of 1 μg Kg^−1^ rat. Group VI (AR + IED + Sid5) received pure Sid intraperitoneally twice a week at a dose of 5 μg Kg^−1^ rat. The animals were given deionized water ad libitum during iron-deficient anemia induction, while ordinary tap water was offered to drink throughout the recovery period. Rats were anesthetized and slaughtered, and their spleen and liver were taken and weighed before the liver was perfused with physiological saline to eliminate blood at the end of the experiment. The relative weight of organs was calculated using the following equation:

(1) $$\mathrm{The}\;\mathrm{relative}\;\mathrm{weight}\;\mathrm{of}\;\mathrm{the}\;\mathrm{organ}=\frac{\mathrm{Weight}\;\mathrm{of}\;\mathrm{the}\;\mathrm{organ}}{\mathrm{Weight}\;\mathrm{of}\;\mathrm{the}\;\mathrm{rat}}\times \;100$$

#### 2.4.3. Measurement of Hematological Parameters

Five hundred microliters of blood were collected in 1.5 mg mL^−1^ purple capped tubes incorporated with disodium salt of ethylenediaminetetraacetic acid (EDTA-2Na) from the tail vein of rats at the end of the 0, 2nd, and 4th week. The hemoglobin level (g dL^−1^) and hematocrit (%) were analyzed on the day of blood collection using CBC (Mindray Vet 28000, China). The plasma iron (μg dL^−1^) was determined using a photometric–colorimetric test based on the chromazurol B (CAB) protocol according to Garčic [49], and ferritin concentration (ng mL^−1^) was determined using an enzyme immunoassay test kit according to White et al. [50]. The total iron-binding capacity (TIBC, μg dL^−1^) was determined using an in vitro quantitative kit (TIBC-LS, MG, STC, USA). At the same time, serum transferrin iron saturation (TSAT %) values were calculated using the following equations:

TSAT (%) = PIC (μg dL^−1^)/TIBC (μg dL^−1^) × 100 (2)

### 2.5. Statistical Analysis

The statistical analysis was conducted using the SPSS program (ver. 23), applying the experimental design variance analysis using one-way ANOVA for growth parameters, food intake, feed efficiency, liver, kidney, and spleen weight, and iron concentration. In contrast, two-way ANOVA was applied for hematological parameters, iron concentration, transferrin saturation %, and ferritin concentration in plasma. The significance level was 0.05, and Tukey’s test was applied following Steel et al. [51].

## 3. Results

### 3.1. Production, Isolation, Purification, and Identification of Catechol-Type Sid from S. tricolor HM10

**Figure 1:**
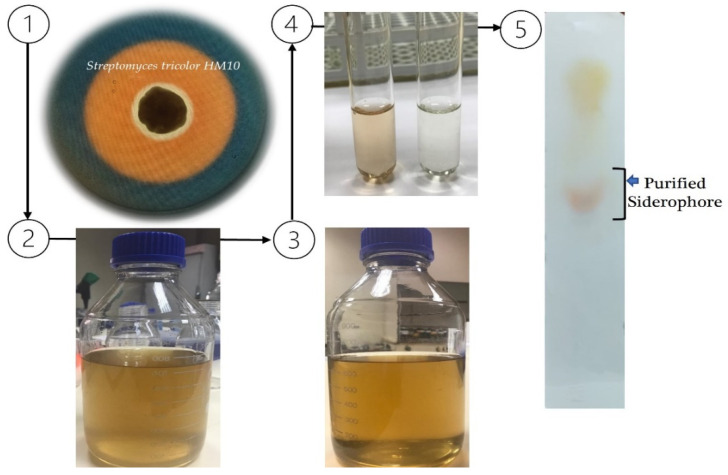
Production, isolation, and purification diagram of catechol-type Sid from *S. tricolor* strain HM10. 1: detection of Sid production on King’s B medium supplemented with Chrome Azurol S (CAS); the orange zone refers to produced Sid, 2: production of Sid in King’s B broth medium, 3: clear supernatant containing produced Sid, 4: detection of catechol-type Sid by Arnow’s Assay [41], and 5: confirming the purity of purified Sid using thin-layer chromatography (TLC).

The *S. tricolor* HM10 strain (Accession: MN527236) was used for Sid production, which was first detected on King’s B agar medium (Figure 1, step 1). Later, mass production on King’s B broth medium at 28 C with contentious agitation for 72 h (step 2) was carried out. Around 50–60 mg Sid L^−1^ medium could be produced using optimal growth conditions. Afterward, the culture broth was cleaned of bacterial cells (step 3). Sid detection and type determination in clear supernatant was achieved according to Arnow’s method (step 4). Only 25–30 mg L^−1^ could be isolated and purified by applying the established protocol using acidified supernatant [43]. To test the purity of the isolated Sid, an appropriate sample was loaded onto a cellulose plate, and one-dimensionally separated, resulting in one single visible band, as shown in Figure 1 (step 5).

### 3.2. Catechol-Type Siderophore Pathway

**Figure 2:**
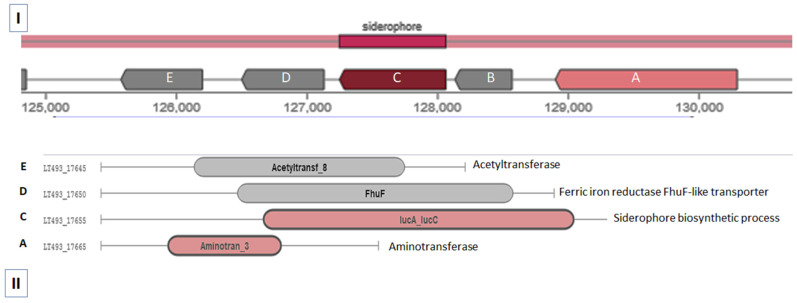
The proposed catechol-type Sid in *S. tricolor* HM10. (**I**) represents Sid pathway in contigs 28 in the genome, (**II**) represents genes involved in Sid production; A, aminotransferase; B, iron transporter; C, IucA/IucC family protein; D, ferric iron reductase FhuF, and E, acetyltransferase.

A putative pathway for catechol-type Sid was identified by searching the sequenced genome of *S. tricolor* HM10. The proposed pathway consisted of the gene that encodes diaminobutyrate-2-oxoglutarate transaminase family protein (gene A), iron transporter (gene B), the IucA/IucC family protein for siderophore biosynthesis (gene C), ferric iron reductase FhuF-like transporter (gene D), and the acetyltransferase (GNAT) domain (gene E). The proposed pathway consisted of five genes with predicted Enterochelin-like Sid production (Figure 2).

### 3.3. TPC and Relative Antioxidant Capacities of Purified Sid from S. tricolor HM10

The TPC and potential antioxidant capacities of purified Sid from *S. tricolor* levels were determined. The TPC in Sid was 452.12 ± 5.78 mg GAE g^−1^, which presented 85.18 ± 4.21 and 97.38 ± 2.95 mmol of TE g^−1^ for DPPH-RSA- and ABTS-RSA-based antioxidant capacities, respectively.

### 3.4. Weight Gain, Food Intake, and Food Efficiency Ratio

Table 2 shows the final body weight, weight gain, food intake, and food efficiency ratio in the rats during the recovery period compared to the SD group. The highest body weight gain was recorded in the SD group, while the lowest body weight gain was recorded in the AR group. The AR group fed an SD with different concentrations of injected Sid exhibited significant changes in weight gain. Remarkably, the AR group fed an SD with 5 μg Kg^−1^ showed the highest weight gain. There was no significant difference with ARs provided an IED with 5 μg Kg^−1^ of Sid. However, feeding ARs with an IED containing double iron content did not significantly affect weight gain compared to AR + SD + Sid5. Notably, high Sid concentration is a key promoter in increasing weight gain. Consequently, the highest food intake was observed with the SD, followed by ARs, which were fed the SD diet and injected with 5 μg Kg^−1^ of Sid, while the lowest food intake was seen in the AR group. Feed efficiency significantly increased when feeding an SD in the SD group compared to the AR group. A significant change was noted with ARs provided an SD or IED with Sid injection. The highest feed efficiency was observed in the AR + SD + Sid5 group, which did not significantly differ from the SD group.

### 3.5. Liver, Kidney, and Spleen Weight and Liver Iron Concentration

The relative weights of the liver, kidneys, and spleen and liver iron concentration (mg g^−1^) are shown in Table 3. The relative weights of the liver and kidneys varied significantly across all groups. The AR group had the highest liver weight among all groups, with a significant difference. Compared to the SD group, the AR + SD and AR + IED groups with 1 or 5 μg Kg^−1^ of Sid showed no significant differences. Consequently, ARs had significantly higher relative liver weights than AR groups fed an SD or IED and injected with 1 or 5 μg Kg^−1^ of Sid. Diet and Sid concentration were clearly related to the relative weight of the organ, and reductions in relative organ weights took place in a dose-dependent manner. The SD and AR groups demonstrated that an IDD affected the relative kidney weight. However, there was no significant difference in relative kidney weight between ARs fed an SD or IED with 1 and 5 μg Kg^−1^ of Sid groups and SD groups. The SD group had the highest relative spleen weight, which differed significantly from the AR group. ARs fed an SD or IED with 1 or 5 μg Kg^−1^ Sid did not display significantly different results from the SD and AR + SD groups. The LIC was significantly lower in the AR group than in the SD group. Among iron-deficient groups, the values observed in the AR + IED + Sid1 and AR + IED + Sid5 groups were considerably higher than those observed in the AR + SD + Sid1 and AR + SD + Sid5 groups or in the AR + SD group.

### 3.6. Measurement of Hematological Parameters of Blood

The hematological parameters, such as RBC (10^12^ L^−1^), HGB (g dL^−1^), HCT (%), MCV (fL), MCH (pg), and MCHC (g dL^−1^), are determined through complete blood counts (CBCs) for blood samples taken at the start (day 0) and at the end of the recovery period (day 28) for different experimental groups are illustrated in Table 4. Immediately after inducing anemia, rats were divided into six boxes with eight rats each, and 0.35 mL of fresh blood was taken from the tail vein. The RBC, HGB, HCT, MCV, MCH, and MCHC were determined through CBCs. The results on day 0 presented the means of the hematological parameters for each group. However, no considerable variation could be seen among AR divided groups regarding RBC, HGB, HCT, MCV, MCH, and MCHC. Of course, the SD group differed significantly from other AR groups. After 14 days, RBC, HGB, HCT, MCV, MCH, and MCHC values were improved considerably due to the attenuation of induced anemia compared to SD or AR groups. Quick enhancement was seen in ARs fed an IED with Sid injection compared to ARs fed an SD with Sid injection.

After 28 days, a non-significant difference was found in the SD group regarding all hematological parameters except RBC. After 28 days, a non-significant reduction in all hematological parameters was found in the AR group except the MCHC value compared to results recorded on day 0. On the contrary, significant changes were found in all parameters in all experimental groups, confirming the efficiency of the anemia recovery treatments.

The RBC count exhibited substantial increases in the group of ARs fed SD. There was no significant difference between ARs that provided an SD or IED with 1 and 5 μg Kg^−1^ Sid and the SD group. The group of ARs provided an SD with Sid injection at 1 and 5 μg Kg^−1^, which resulted in significant HBG increases compared to AR + SD or AR groups. Accordingly, HCT % exhibited a considerable increase in the blood of ARs fed an SD with 1 and 5 μg Kg^−1^ than the group of ARs fed an SD. Although there was a significant difference between SD- and AR-treated groups, the HCT % in the treated group indicated high iron absorption rates depending on the treatment. The MCV of RBC showed a significant difference between the AR group and AR-treated groups. Most of the monitored hematological parameters observed when providing an SD in the presence of Sid injection were efficiently better than those observed when providing an SD without Sid injection or even an IED with Sid injection.

In contrast, feeding an SD or IED with Sid helped improve the RBC status and attenuate the MCV compared with the SD group. Consequently, the MCH and MCHC values underwent a considerable improvement in hemoglobin content in the RBC of ARs fed an SD with 1 and 5 μg Kg^−1^ than the group of ARs fed an SD, which indicated a normal status compared to the SD group.

### 3.7. Plasma Iron Concentration, Transferrin Saturation %, and Ferritin Concentration in Plasma

**Figure 3:**
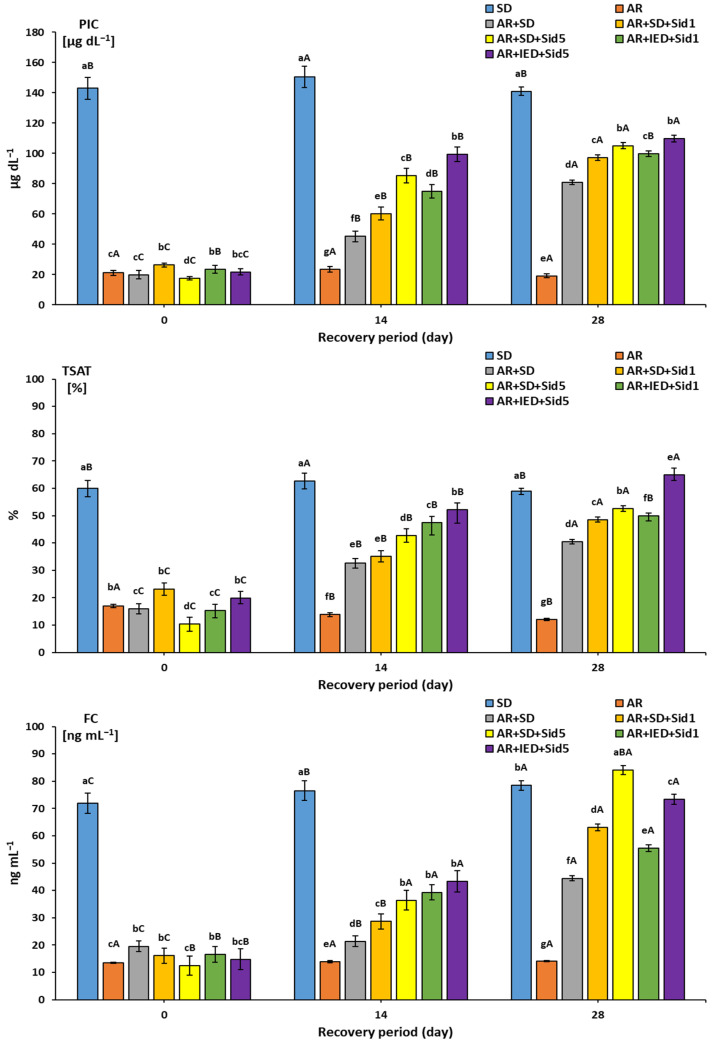
Plasma iron concentration (PIC), transferrin saturation % (TSAT), and ferritin concentration (FC) in rats fed different formulated experimental diets during a recovery period of up to 28 days (*n* = 8). SD: standard diet, AR: anemic rat fed iron-deficient diet, AR + SD: AR fed SD, AR + SD + Sid1: AR fed SD and intraperitoneally injected with Sid at 1 μg Kg^−1^ rat twice per week, AR + SD + Sid5: AR fed SD and intraperitoneally injected with Sid at 5 μg Kg^−1^ rat twice per week. AR + IED + Sid1: AR fed iron-enriched diet (IED) and intraperitoneally injected with Sid at 1 μg Kg^−1^ rat twice per week, AR + IED + Sid5: AR fed IED diet and intraperitoneally injected with Sid at 5 μg Kg^−1^ rat twice per week, ^a,b,c,d,e,f,g^: bars for the same recovery day not sharing similar letters are significantly different (*p* > 0.05), ^A,B,C^: bars for each treatment not sharing similar letters during the recovery period are significantly different (*p* > 0.05).

Figure 3 shows the plasma iron levels, transferrin saturation %, and ferritin concentration in different experimental groups. At the beginning of the recovery period, the PIC values of the SD and iron-deficient diet groups were 143.83 μg dL^−1^ and (17.68–26.30 μg dL^−1^), respectively. Significant increases were seen during the recovery period when ARs were fed an SD or an SD or IED with 1 or 5 μg Kg^−1^ Sid. The PIC values in the AR + SD group were significantly lower than in the other four groups on days 14 and 28. Significant increases were observed in AR + SD + Sid5 and AR + IED + Sid5 groups on day 14 and day 28 compared to the values for the AR + SD + Sid1 and AR + IED + Sid1 groups. The TSAT levels in the SD and AR groups were 59.93% and (10.34–19.84%), respectively, at the beginning of the recovery period. No significant difference was recorded in the SD group until the end of the recovery period. On the contrary, the TSAT in the AR group was significantly decreased with time progression. At the beginning of the recovery period, ferritin concentration in the SD and AR blood serum was 71.92 and (12.48–19.56) ng mL^−1^ (Figure 3).

No significant difference was recorded in the AR group until the end of the recovery period. On the contrary, the FC in the SD group was significantly increased with time progression. In comparison, the most significant increases were noticed in ARs fed an IED with 5 μg Kg^−1^ Sid on days 14 and 28.

## 4. Discussion

Siderophores are secondary metabolites produced by various organisms to scavenge iron, thereby increasing its bioavailability and absorption into the cell. They are characterized by a high affinity for ferric iron, forming powerful iron-chelating complexes [14,18]. Siderophores recently received considerable attention, and many studies regarding their application in medicine and agriculture have been carried out [14,19,20,21,22,23,24]. Different synthesized siderophores have been identified, including catecholate-type, hydroxamate-type, carboxylate-type, and mixed-type siderophores, as reviewed by Sah and Singh [52]. The production and isolation of catecholate-type Sid from *S. tricolor* HM10 were previously established [39]. In the current research, a catechol-type Sid was successfully isolated and purified from the *S. tricolor* HM10 strain in significant quantities as 25–30 mg Sid L^−1^ medium under optimal conditions. The obtained results agree with the produced and purified siderophores in previous studies [43,53,54,55].

The proposed catechol-type siderophore pathway was identified based on genome sequencing and bioinformatic tools, i.e., antiSMASH online software. The intended pathway contains five genes similar to the petrobactin biosynthetic gene cluster from *Bacillus anthracis*. This pathway is composed of *asb* locus (*asbABCDEF*) and activates catecholate siderophore production [56]. The absB protein demonstrated substrate positions in the binding pocket and reaction flexibility [57]. *Bacillus anthracis* can produce two catechol-type siderophores (petrobactin and bacillibactin) under iron-limited conditions. Bacillibactin siderophores are highly sensitive to iron concentration, whereas petrobactin is less dependent on Fe [58]. Catechol-type siderophores are formed from 3,4-dihydroxybenzoyl spermidine (3,4-DHB-SPD) via the condensation process, followed by an activated form of citrate to produce 4-dihydroxybenzoyl spermidinyl citrate (3,4-DHB-SPD-CT). Both previously produced components condensate to form a mature siderophore [56]. Our study identified the expected catechol-type siderophore molecular weight to be 658.33 (M). This siderophore is similar close to Enterochelin (MW = 668.5), which can be produced from *E. coli* [59] and *Pseudomonas aeruginosa* [60]. Iron plays an essential role in maintaining normal human metabolism and is necessary for most organisms [2]. As is evidently known, iron-deficiency-induced anemia could cause a low hemoglobin concentration and a deficiency of iron enzymes, which initiates systemic dysfunction and abnormal macrophage secretion, further damaging immune function [61,62,63]. IDA could lead to numerous pathologies, particularly delays in development and behavior among children, girls, and pregnant women [3,4,5]. Interestingly, the isolated Sid in the current study expressed valuable TPC with potential antioxidant capacities against DPPH and ABTS radical scavenging activity, adding additional features to the Sid. Indeed, phenolic content and relative antioxidant capacity revealed that catecholate-type Sid has potential antioxidant activity [64,65].

The rats’ final body weight, weight gain, food intake, and food efficiency ratio compared to the SD group during the recovery period were carefully investigated. During the recovery period, the AR group fed an SD and ARs fed an SD with different concentrations of injected Sid exhibited significant changes in weight gain. It could be noticed that Sid injection improved the weight gain in a dose-dependent manner in the presence of available iron in the diet. The increases in the weight gain might have been due to improved metabolism and increased fat and muscle mass, which was observed in the feed efficiency ratio [35]. Remarkably, the AR group fed an SD with 5 μg Kg^−1^ from Sid showed the highest weight gain. Although AR groups were fed an IED containing double iron content, the weight gain was not significantly changed. It might be because a diet containing high inorganic salt such as ferric citrate had side effects on the stomach and affected appetite and digestion [66]. Feed efficiency significantly increased with the feeding of an SD in the SD group compared to the AR group. A significant change was noted with ARs fed an SD or IED with Sid injection. The highest feed efficiency was observed in the AR + SD + Sid5 group, which did not significantly differ from the SD group. Notably, Sid concentration is a key promoter in increasing weight gain [35].

Consequently, the relative weights of the liver, kidneys, and spleen were linked to growth and body weight gain. Weight gained in the organs might have been due to improved metabolism and increased fat and muscle mass, as observed and indicated [36,39]. Consequently, ARs with an SD or IED injected with 1 μg Kg^−1^ of Sid had significantly lower relative liver weights than in the AR group, reflecting the improvement in body weight. Indeed, the higher relative weight may explain the higher organ weight and lower body weight. Diet and Sid concentration were clearly related to weight recovery in a dose-dependent manner, similarly noted in [35,38]. IDA resulted in a negatively skewed iron balance, causing a reduction in LIC and increased serum ferritin levels. Subsequently, decreasing iron intake will cause a decrease in the TSAT level and an increase in the TIBC level, resulting in “latent iron deficiency” [38]. In addition, as the iron deficiency state progresses, subjective symptoms of anemia appear, and the HGB level and HCT% decrease, resulting in “iron deficiency anemia” [67]. Indeed, LIC was measured as an index to determine the possible iron deficiency level in the body. On day 0, the PIC concentration confirmed that the iron-deficient diet group induced the latent iron deficiency condition. Among the iron-deficient groups, the values observed in the AR + IED + Sid1 and AR + IED + Sid5 groups were considerably higher than those observed in the AR + SD + Sid1 and AR + SD + Sid5 groups or in the AR + SD group. This may have indicated that iron was more efficiently absorbed and stored in the liver in the presence of both high iron content in the diet and Sid [35,38]. This relationship is directly related to serum transferrin iron saturation (TSAT %), which increased associatively (Figure 2) and was confirmed [38].

Surprisingly, a noticeable improvement in the hematological parameters such as RBC, HGB, HCT, MCV, MCH, and MCHC was observed. Immediately after inducing anemia, the RBC, HGB, HCT, MCV, MCH, and MCHC were monitored every two weeks up to 4 weeks. Significant changes were found in all parameters in all experimental groups, confirming the efficiency of the anemia recovery treatments. After 2 weeks, the remarkable improvements in HGB, HCT%, MCV, and MCHC in treated AR were evident in the efficiency of Sid in the presence of iron in the diet to attenuate anemia. This may be because Sid could anchor a specific gradient outside the cell membrane, depending on membrane affinity and length, thereby enhancing the ability to capture iron from surrounding matrixes [20,21]. The above results clarified that providing an SD to AR groups with Sid injection at 1 and 5 μg Kg^−1^ resulted in more significant improvements in the hematological parameters than feeding an SD alone or even an IED with Sid injection. Accordingly, considerable increases in HGB, HCT%, MCV, MCH, and MCHC values promoted recovery from iron-deficiency-induced anemia. Iron is mainly absorbed in the duodenum; there are two types of iron in the diet: non-heme and heme iron, each with a different absorption mechanism. Non-heme iron is taken up into intestinal epithelial cells by the divalent metal transporter 1 (DMT1), though the iron in the intestinal tract would then need to be solubilized [68]. However, acidified digesta by gastric acid is gradually neutralized by pancreatic and intestinal juice in the duodenum, and poorly soluble salts are formed with coexisting phosphoric acid as the pH increases, which results in poor iron absorption [69]. The solubility of the iron citrate used in the tested diet in this study was low, and insoluble salts were formed with an increase in pH. However, the results indicated that the presence of Sid might contribute to enhanced iron absorption [35]. Siderophores have low molecular weights and facilitate the iron acquisition and counter iron deficiency by high-affinity iron (III) ligands [14]. As Sid preferentially chelates iron, ferric iron could be chelated by Sid, increasing iron availability. Later on, when ferric iron is reduced to ferrous iron, Sid will release it. This is a crucial strategy for increasing iron absorption [14,70]. The role of siderophores is to dissolve ferric iron and transport it into the cytoplasm, so siderophores have the potential to become a nutritional fortifier [20].

As previously hypothesized, Sid could anchor a specific gradient outside the cell membrane, depending on membrane affinity and length, thereby enhancing the ability to capture iron from surrounding matrixes [20,21]. Iron is an indispensable nutrient; however, excess iron works as a pro-oxidant to produce hydroxyl radicals. Therefore, iron absorption has a regulatory system [71,72]. There are two iron transporters in epithelial cells, namely DMT1, an importer of iron, and feroportin (Fpn), an exporter of iron. The expression of hepcidin, a peptide hormone produced in the liver, is promoted during iron deficiency. Blood hepcidin binds to Fpn, which accelerates the internalization of the complex and the degradation of Fpn. Thus, the exportation of iron from enterocytes to ferritin is reduced. An inverse relationship between hepcidin expression and iron absorption and the expression of duodenal iron transporters was reported in rats [73]. These might be reasonable explanations for the significant increases observed in the PIC of ARs fed an SD or IED with 1 or 5 μg Kg^−1^ Sid. Likewise, the TSAT was associated significantly with serum iron (SI), as evidently presented [35,38]. Significant increases in ferritin concentration were noticed in ARs fed an IED with 5 μg Kg^−1^ Sid on days 14 and 28. On the other hand, indigestible saccharides, such as water-soluble dietary fibers and oligosaccharides, are reportedly fermented in the large intestine by intestinal bacteria to produce short-chain fatty acids such as acetic acid, propionic acid, n-butyric acid, or organic acids such as lactic acid and succinic acid. These acids acidify the lumen, increase the number of minerals solubilized in the intestine, and promote iron absorption in the intestine [74,75,76].

Indeed, in small species, intraperitoneal injection is used instead of intravenous access, and it can be used to safely administer large volumes of fluid. A higher affinity between siderophores and iron than between transferrin and iron is recognized. The catecholate-type siderophore has stronger iron affinities than transferrin in comparison to other categories of siderophores. A siderophore–iron complex is expected to enter the cell through simple diffusion (according to the low iron concentration inside the cells regarding anemia induced by iron deficiency) via the endocytosis of the transferrin receptor (TfR1) Siderocalin/Lipocalin 2/Neutrophil Gelatinase Associated Lipocalin, which is an innate immune system protein with bacteriostatic activity, or ATP-binding cassette (ABC) transporters, the energy-dependent efflux transporters. Red pulp macrophages (RPMs) and Kupffer cells (KCs) express complete machinery for RBC clearance and heme iron recycling [77,78,79,80,81,82].

Noticeably, at present, we did not delineate the detailed mechanism of these changes, and further experiments are needed to elucidate the exact underlying mechanisms. Combing our results with clearly reviewed studies, we suggest that the mechanism of action of Sid is to be effective in chelating iron in the entire intestine and binding to the cell membrane and will then find a way to transfer iron elements into the cell. However, to further confirm this hypothesis, we need to design a signaling experiment to elucidate iron pathways. In addition, the possibility of excess iron absorption needs to be examined using an iron-repleted diet and prolonged feeding conditions.

## 5. Conclusions

Isolated Sid from *S. tricolor* HM10 was evaluated through an anemia-induced rat model to study its potential to accelerate recovery from IDA. This study revealed that Sid significantly improved weight gain and iron-deficiency-induced anemia was effective during recovery from anemia and could be a practical iron-nutritive fortifier. Combining our findings with previously mentioned studies, we suggest that Sid’s mechanism of action is effective in chelating iron in the entire intestine, binding to the cell membrane, and then finding a way to transfer iron elements into the cell. Noticeably, at present, we did not delineate the detailed mechanism of these changes, and the design of a signaling experiment to confirm this hypothesis is highly recommended. In addition, the possibility of excess iron absorption needs to be examined using an iron-repleted diet and prolonged feeding conditions.

## Figures and Tables

**Table 1:** Composition of the experimental diets used in iron-deficiency anemia induction and control (g 100 g^−1^ diet).

Ingredients	Anemia-Inducing Period		Experimental Diet and Anemia Recovery Period					
	SD ^a^	IDD	AR	AR + SD	AR + SD + Sid1	AR + SD + Sid5	AR + IED + Sid1	AR + IED + Sid5
Corn starch	53.43	53.45	53.45	53.43	53.43	53.43	53.41	53.41
Casein	20.00	20.00	20.00	20.00	20.00	20.00	20.00	20.00
Sucrose	10.00	10.00	10.00	10.00	10.00	10.00	10.00	10.00
Soybean oil	7.00	7.00	7.00	7.00	7.00	7.00	7.00	7.00
Cellulose powder	5.00	5.00	5.00	5.00	5.00	5.00	5.00	5.00
L-Cystine	0.30	0.30	0.30	0.30	0.30	0.30	0.30	0.30
Choline bitartrate	0.25	0.25	0.25	0.25	0.25	0.25	0.25	0.25
AIN-93 vitamin mixture	1.00	1.00	1.00	1.00	1.00	1.00	1.00	1.00
AIN-93G mineral mixture ^b^	1.75	1.75	1.75	1.75	1.75	1.75	1.75	1.75
Calcium carbonate	1.25	1.25	1.25	1.25	1.25	1.25	1.25	1.25
Ferric citrate	0.02	–	–	0.02	0.02	0.02	0.04	0.04
Sid (μg Kg^−1^ Rat) ^c^	–	–	–	–	1 μg	5 μg	1 μg	5 μg

^a^ Standard diet (SD) used for adaptation, the iron content per 100 g of diet: 4.50 mg, iron-deficient diet (IDD); 0.14 mg, iron-enriched diet (IED); 9.00 mg, ^b^ iron-free mineral mixture; ^c^ pure Sid was intraperitoneally injected twice per week as (μg Kg^−1^ rat).

**Table 2:** Growth parameters, food intake, and feed efficiency of rats fed different formulated experimental diets with an intraperitoneal injection of Sid after a recovery period of 4 weeks (*n* = 8).

Items	Experimental Groups						
	SD	AR	AR + SD	AR + SD + Sid1	AR + SD + Sid5	AR + IED + Sid1	AR + IED + Sid5
Initial BW (g) *	178.24 ± 7.21	168.00 ± 11.67	187.00 ± 7.78	179.83 ± 6.94	176.50 ± 5.10	171.33 ± 5.77	174.83 ± 3.89
Final BW (g)	271.45 ± 6.48	185.50 ± 14.73	236.67 ± 4.24	246.33 ± 6.14	255.83 ± 5.51	238.67 ± 5.02	251.67 ± 6.95
BW gain (g)	93.21 ± 5.75 ^a^	12.50 ± 4.91 ^e^	49.87 ± 2.83 ^d^	66.50 ± 4.09 ^c^	79.33 ± 3.68 ^b^	67.33 ± 3.35 ^c^	76.83 ± 4.63 ^b^
Food intake (g day^−1^) ^#^	19.39 ± 1.76 ^a^	12.03 ± 1.01 ^d^	15.78 ± 1.63 ^c^	16.42 ± 1.74 ^bc^	17.06 ± 1.84 ^b^	15.91± 1.65 ^c^	16.11 ± 1.69 ^bc^
Feed efficiency ^##^	0.172 ± 0.015 ^a^	0.074 ± 0.004 ^e^	0.112 ± 0.013 ^d^	0.145 ± 0.015 ^bc^	0.166 ± 0.017 ^a^	0.151 ± 0.013 ^bc^	0.148 ± 0.017 ^bc^

SD: standard diet, AR: anemic rat fed iron-deficient diet, AR + SD: AR fed SD, AR + SD + Sid1: AR fed SD and intraperitoneally injected with Sid at 1 μg Kg^−1^ rat twice per week, AR + SD + Sid5: AR fed SD and intraperitoneally injected with Sid at 5 μg Kg^−1^ rat twice per week. AR + IED + Sid1: AR fed iron-enriched diet (IED) and intraperitoneally injected with Sid at 1 μg Kg^−1^ rat twice per week, AR + IED + Sid5: AR fed IED diet and intraperitoneally injected with Sid at 5 μg Kg^−1^ rat twice per week, *: initial BW was recorded at the beginning of the experiment, BW: body weight, ^#^: calculated as gram diet/rate per day, ^##^ feed efficiency = BW gain/food intake, ^a,b,c,d,e^: there is no significant difference (*p* > 0.05) between any two means within the same row with the same superscripted letters.

**Table 3:** The relative weight of liver, kidneys, and spleen and iron concentration in the liver of rats fed different formulated experimental diets with an intraperitoneal injection of Sid after a recovery period of 4 weeks (*n* = 8).

Items	Experimental Groups						
	SD	AR	AR + SD	AR + SD + Sid1	AR + SD + Sid5	AR + IED + Sid1	AR + IED + Sid5
Relative liver weight (%)	3.45 ± 0.12 ^bc^	3.85 ± 0.14 ^a^	3.56 ± 0.06 ^b^	3.56 ± 0.05 ^b^	3.62 ± 0.09 ^b^	3.42 ± 0.07 ^c^	3.60 ± 0.05 ^b^
Relative kidney weight (%)	0.70 ± 0.08 ^b^	0.89 ± 0.02 ^a^	0.79 ± 0.05 ^b^	0.73 ± 0.01 ^b^	0.72 ± 0.02 ^b^	0.72 ± 0.02 ^b^	0.73 ± 0.03 ^b^
Relative spleen weight (%)	0.32 ± 0.02 ^b^	0.39 ± 0.02 ^a^	0.34 ± 0.02 ^b^	0.31 ± 0.02 ^b^	0.32 ± 0.01 ^b^	0.30 ± 0.02 ^b^	0.30 ± 0.03 ^b^
LIC (mg g^−1^ liver)	98.12 ± 3.48 ^a^	11.24 ± 1.89 ^e^	35.24 ± 4.18 ^d^	46.21 ± 3.24 ^c^	48.54 ± 3.28 ^c^	64.25 ± 4.19 ^b^	69.24 ± 4.28 ^b^

SD: standard diet, AR: anemic rat fed iron-deficient diet, AR + SD: AR fed SD, AR + SD + Sid1: AR fed SD and intraperitoneally injected with Sid at 1 μg Kg^−1^ rat twice per week, AR + SD + Sid5: AR fed SD and intraperitoneally injected with Sid at 5 μg Kg^−1^ rat twice per week. AR + IED + Sid1: AR fed iron-enriched diet (IED) and intraperitoneally injected with Sid at 1 μg Kg^−1^ rat twice per week, AR + IED + Sid5: AR fed IED diet and intraperitoneally injected with Sid at 5 μg Kg^−1^ rat twice per week, LIC: liver iron concentration, ^a,b,c,d,e^: there is no significant difference (*p* > 0.05) between any two means within the same row with the same superscripted letters.

**Table 4:** Hematological parameters of rats fed different formulated experimental diets with an intraperitoneal injection of Sid at 1 and 5 μg Kg^−1^ at starting and recovery period up to 28 days (*n* = 8).

Hematological Parameters	Days	Experimental Groups						
		SD	AR	AR + SD	AR + SD + Sid1	AR + SD + Sid5	AR + IED + Sid1	AR + IED + Sid5
RBC [10^12^ L^−1^]	0	6.26 ± 0.08 ^aC^	2.68 ± 0.17 ^bA^	2.57 ± 0.10 ^bC^	2.30 ± 0.41 ^bC^	2.67 ± 0.15 ^bC^	2.57 ± 0.15 ^bC^	2.69 ± 0.24 ^bC^
	14	6.57 ± 0.27 ^aB^	2.47 ± 0.24 ^dA^	3.27 ± 0.31 ^cB^	4.18 ± 0.75 ^bB^	4.77 ± 0.59 ^bB^	4.61 ± 0.09 ^bB^	5.37 ± 0.81 ^bB^
	28	8.20 ± 0.31 ^aA^	2.41 ± 0.15 ^cA^	6.52 ± 0.19 ^bA^	7.21 ± 0.25 ^aA^	7.80 ± 0.54 ^aA^	7.23 ± 0.30 ^aA^	7.37 ± 0.27 ^aA^
HGB [g dL^−1^]	0	15.90 ± 0.35 ^aA^	7.06 ± 1.06 ^abA^	7.88 ± 2.09 ^abB^	7.52 ± 1.01 ^bC^	6.52 ± 0.47 ^bC^	6.43 ± 0.20 ^bC^	6.87 ± 0.25 ^bC^
	14	16.13 ± 0.47 ^aA^	6.91 ± 0.92 ^eA^	8.98 ± 0.87 ^dB^	9.23 ± 0.84 ^dB^	12.24 ± 1.18 ^bcB^	11.08 ± 0.79 ^cB^	13.01 ± 0.64 ^bB^
	28	16.60 ± 0.22 ^aA^	6.36 ± 0.95 ^dA^	13.58 ± 1.42 ^bcA^	15.43 ± 0.74 ^abA^	16.18 ± 0.85 ^aA^	14.88 ± 0.30 ^bA^	15.15 ± 0.51 ^abA^
HCT [%]	0	36.05 ± 2.86 ^aA^	13.69 ± 1.06 ^bA^	11.63 ± 0.46 ^bC^	10.12 ± 1.81 ^bC^	11.93 ± 0.58 ^bC^	11.60 ± 0.87 ^bC^	14.70 ± 0.84 ^bC^
	14	36.78 ± 1.13 ^aA^	13.74 ± 0.79 ^dA^	13.15 ± 0.91 ^dB^	13.14 ± 0.97 ^dB^	14.79 ± 1.47 ^cB^	15.27 ± 0.59 ^bcB^	17.25 ± 1.51 ^bB^
	28	38.60 ± 0.48 ^aA^	12.33 ± 0.95 ^cA^	21.30 ± 0.57 ^bA^	24.00 ± 0.94 ^aA^	25.10 ± 1.07 ^aA^	23.10 ± 0.72 ^abA^	23.20 ± 0.78 ^abA^
MCV [fL]	0	91.30 ± 0.74 ^aA^	15.38 ± 0.25 ^bA^	65.13 ± 0.04 ^bA^	74.72 ± 0.77 ^bA^	84.95 ± 0.69 ^bA^	65.08 ± 0.38 ^bA^	68.28 ± 0.58 ^abA^
	14	90.77 ± 0.92 ^aA^	13.91 ± 0.54 ^eA^	55.78 ± 0.25 ^bB^	48.17 ± 1.81 ^cB^	40.80 ± 1.29 ^dB^	49.27 ± 2.47 ^cB^	39.75 ± 3.19 ^dB^
	28	92.08 ± 0.67 ^aA^	13.84 ± 0.22 ^bA^	52.88 ± 1.96 ^aC^	33.40 ± 0.83 ^aC^	32.23 ± 1.64 ^aC^	32.32 ± 1.49 ^aC^	31.48 ± 1.87 ^aC^
MCH [pg]	0	29.08 ± 2.80 ^aA^	7.00± 0.52 ^bA^	8.47 ± 1.93 ^bC^	6.67 ± 0.20 ^bC^	6.76 ± 0.14 ^bC^	6.94 ± 0.20 ^bC^	7.11 ± 0.47 ^bC^
	14	28.75 ± 0.19 ^aA^	7.08 ± 0.49 ^dA^	12.57 ± 2.01 ^bcB^	14.17 ± 0.93 ^bcB^	15.34 ± 1.28 ^bB^	15.07 ± 0.68 ^bB^	15.08 ± 1.27 ^bB^
	28	30.25 ± 0.89 ^aA^	6.30 ± 0.47 ^cA^	20.80 ± 1.13 ^bA^	21.38 ± 0.41 ^bA^	20.55 ± 0.79 ^bA^	20.72 ± 0.49 ^bA^	20.55 ± 1.05 ^bA^
MCHC [g dL^−1^]	0	31.71 ± 1.27 ^aA^	6.85 ± 0.42 ^aB^	8.43 ± 1.92 ^aB^	6.82 ± 0.16 ^aB^	6.82 ± 0.35 ^aB^	6.94 ± 0.32 ^aB^	5.84 ± 0.22 ^aB^
	14	30.91 ± 2.28 ^aA^	6.71 ± 0.64 ^eAB^	12.80 ± 1.52 ^dA^	15.98 ± 0.86 ^cA^	19.72 ± 0.98 ^bA^	16.74 ± 1.65 ^cA^	21.76 ± 2.15 ^bA^
	28	33.75 ± 1.66 ^aA^	6.17 ± 0.38 ^bA^	31.60 ± 0.99 ^aA^	32.20 ± 1.03 ^aA^	34.10 ± 1.46 ^aA^	31.42 ± 1.51 ^aA^	32.55 ± 1.39 ^aA^

SD: standard diet, AR: anemic rat fed iron-deficient diet, AR + SD: AR fed SD, AR + SD + Sid1: AR fed SD and intraperitoneally injected with Sid at 1 μg Kg^−1^ rat twice per week, AR + SD + Sid5: AR fed SD and intraperitoneally injected with Sid at 5 μg Kg^−1^ rat twice per week. AR + IED + Sid1: AR fed iron-enriched diet (IED) and intraperitoneally injected with Sid at 1 μg Kg^−1^ rat twice per week, AR + IED + Sid5: AR fed IED diet and intraperitoneally injected with Sid at 5 μg Kg^−1^ rat twice per week, RBC: red blood cells, HGB: hemoglobin, HCT: hematocrit, MCV: mean corpuscular volume, MCH: mean corpuscular hemoglobin, MCHC: mean corpuscular hemoglobin concentration, ^a,b,c,d,e^: there is no significant difference (*p* > 0.05) between any two means within the same row that have the same superscripted letters, ^A,B,C^: there is no significant difference (*p* > 0.05) between any two means for the same attribute within the same column with the same superscripted letters.

## Data Availability

The data presented in this study are available on request from the corresponding author.

## Abbreviations

2,3-DHBA	2,3-Dihydroxybenzoic acid
ABC	ATP-binding cassette transporters
ABTS	2,2′-azino-bis(3-ethylbenzothiazoline-6-sulfonic acid)
AOA	Antioxidant activity
AR	Anemic rat
AR + IED + Sid1	Anemic rat fed iron-enriched diet with an intraperitoneal injection of 1 μg Sid Kg^−1^
AR + IED + Sid5	Anemic rat fed iron-enriched diet with an intraperitoneal injection of 5 μg Sid Kg^−1^
AR + SD + Sid1	Anemic rat fed standard diet with an intraperitoneal injection of 1 μg Sid Kg^−1^
AR + SD + Sid5	Anemic rat fed standard diet with an intraperitoneal injection of 5 μg Sid Kg^−1^
ARs	Anemic rats
CAB	Chromazurol B
CAS	Chrome Azurol S
CPCSEA	Purpose of the Control and the Supervision of Experiments on Animals
DPPH	1,1-diphenyl-2-picryl hydrazine
dw	Dry weight
EDTA-2Na	Ethylenediaminetetraacetic acid
FC	Ferritin concentration
GAE	Gallic acid equivalent
HCT	Hematocrit
HGB	Hemoglobin
IAEC	Institutional Animal Ethics Committee
IDA	Iron-deficiency-induced anemia
IDD	Iron-deficient diet
IED	Iron-enriched diet
KC	Kupffer cell
KSA	Kingdom of Saudi Arabia
LIC	Lever’s iron concentration
MBCa	Maltobionic acid calcium salt
MCH	Mean corpuscular hemoglobin
MCHC	Mean corpuscular hemoglobin concentration
MCV	Mean corpuscular volume
NCBE	National Committee of Bioethics
NCBI	National Center for Biotechnology Information
NRs	Normal rats
PIC	Plasma iron concentration
RBC	Red blood cells
RPM	Red pulp macrophage
SD	Standard diet
Sid	Siderophore
TE	Trolox Equivalents
TIBC	Total iron-binding capacity
TLC	Thin-layer chromatography
TPC	Total phenolic compounds
TSAT	Transferrin saturation %
